# Schwannoma as Extraspinal Swelling Over the Thoracolumbar Region: A Tumor at a Rare Site

**DOI:** 10.7759/cureus.58157

**Published:** 2024-04-12

**Authors:** Manu Srinivas, Prabhat Nichkaode, Bijay Sharma, Shriya Haval

**Affiliations:** 1 General Surgery, Dr. D. Y. Patil Medical College, Hospital and Research Centre, Dr. D.Y. Patil Vidyapeeth (Deemed to be University), Pune, IND

**Keywords:** fine-needle biopsy, imaging analysis, thoracolumbar region, extraspinal swelling, schwannoma

## Abstract

This case report describes an unusual presentation of schwannoma, a typically benign and solitary tumor originating from Schwann cells in peripheral nerves. While the literature on extraspinal schwannomas is limited, this report discusses the case of a 21-year-old female with complaint of a back swelling persisting for two years, causing discomfort during sleep. The oval-shaped swelling, measuring 7x6 cm, was located over the T11-T12-L1 vertebrae, with normal overlying skin, pinchable hardness, and fixation to the vertebrae. The patient had no history of pain or weakness in the lower limbs. Fine-needle aspiration cytology (FNAC) yielded inconclusive results. X-ray imaging of the thoracolumbar spine revealed a soft tissue shadow over the T11-T12-L1 vertebrae. The patient underwent complete surgical excision through a vertical incision, emphasizing the importance of preoperative imaging for accurate diagnosis, optimal surgical planning, and ensuring procedural safety.

## Introduction

The cervical cord is the most frequent site for nerve sheath tumors, followed by the thoracic and lumbosacral sections of the spinal cord [1]. Neurogenic tumors arise in tissues derived from the embryonic neural crest and are classified based on whether the tumor cell originates from a nerve sheath, nerve cells (ganglia), paraganglia, or peripheral nerves and can be found throughout the body. Meningiomas and spinal schwannomas are the two most typical spinal nerve sheath tumors [2]. Schwannomas are more frequently found in adulthood and are frequently intradural-extramedullary masses and 90% are benign, spontaneous solitary tumors [3]. In the thorax, neurogenic tumors are most commonly found in the posterior mediastinum, accounting for 75% of all posterior mediastinal neoplasms [4]. Schwannoma has several names in the literature including neurilemmoma, neurilemoma, and neuroma. The majority of patients have modest sensory signs such as unexpected pain or par aesthesia when the nerves are palpated; weakening may occur but is less frequent [5].

Schwannomas exhibit a predilection for the head, neck, and flexor sides of the extremities, with around 45% occurring in the head and neck area. They can originate from various peripheral, cranial, or autonomic nerves and may be associated with neurofibromatosis type II [6]. Representing approximately 30% of all spinal neoplasms, schwannomas demonstrate a slight male-to-female predilection (ratio of 1.23-1.5:1), favoring males [7]. Typically, these tumors are unilocular, cystic, asymptomatic, and benign, being encapsulated and attached to or covered by a nerve. Schwannomas are often solitary, dissecting free while preserving the nerve of origin and appearing to push the nerve axons. Many cases are asymptomatic and incidentally discovered during routine imaging. While schwannomas are commonly found in various anatomical locations, the thoracolumbar region is an exceptionally rare site. This report presents a case of an extremely uncommon extraspinal schwannoma in the thoracolumbar region, emphasizing the need for physicians to be aware of this unusual clinical condition when evaluating patients with similar clinical presentations

## Case presentation

A 21-year-old female presented with a two-year history of swelling on her back, causing discomfort while sleeping on her back. Remarkably, there was no associated pain, and the patient continued with her daily activities without any hindrance. No neurological symptoms were reported, and there was no history of trauma or weight loss. The swelling, initially measuring 2x2 cm, gradually increased over time and reached its present size of 7 x 6 cm.

On clinical examination, an oval-shaped swelling of 7x7 cm was observed over the back, specifically overlying T 11-12, L1 vertebrae. The skin over the swelling appeared normal and pinchable, while the swelling itself was firmly fixed to the vertebrae and presented a hard consistency.

**Figure 1:**
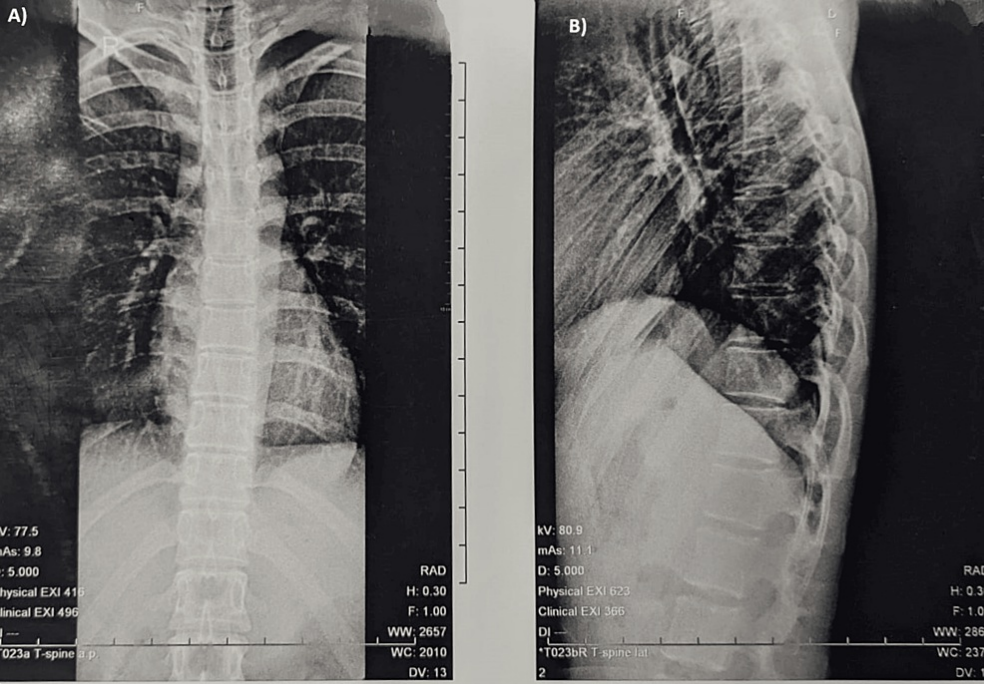
Thoracolumbar spine lateral (A) and anteroposterior (B) view showing soft tissue shadow.

Thoracolumbar region spine X-ray revealed a soft tissue shadow over the T 11-12 and L1 vertebrae (Figure 1). Ultrasonography of the local swelling was done, which exhibited a heterogeneous lesion with posterior acoustic enhancement measuring 24x12x20 mm, displaying some cystic areas and significant internal vascularity noted in the midline over the lower back in the intramuscular plane. Fine needle aspiration cytology (FNAC) yielded inconclusive results, while routine blood investigations were within normal limits.

**Figure 2:**
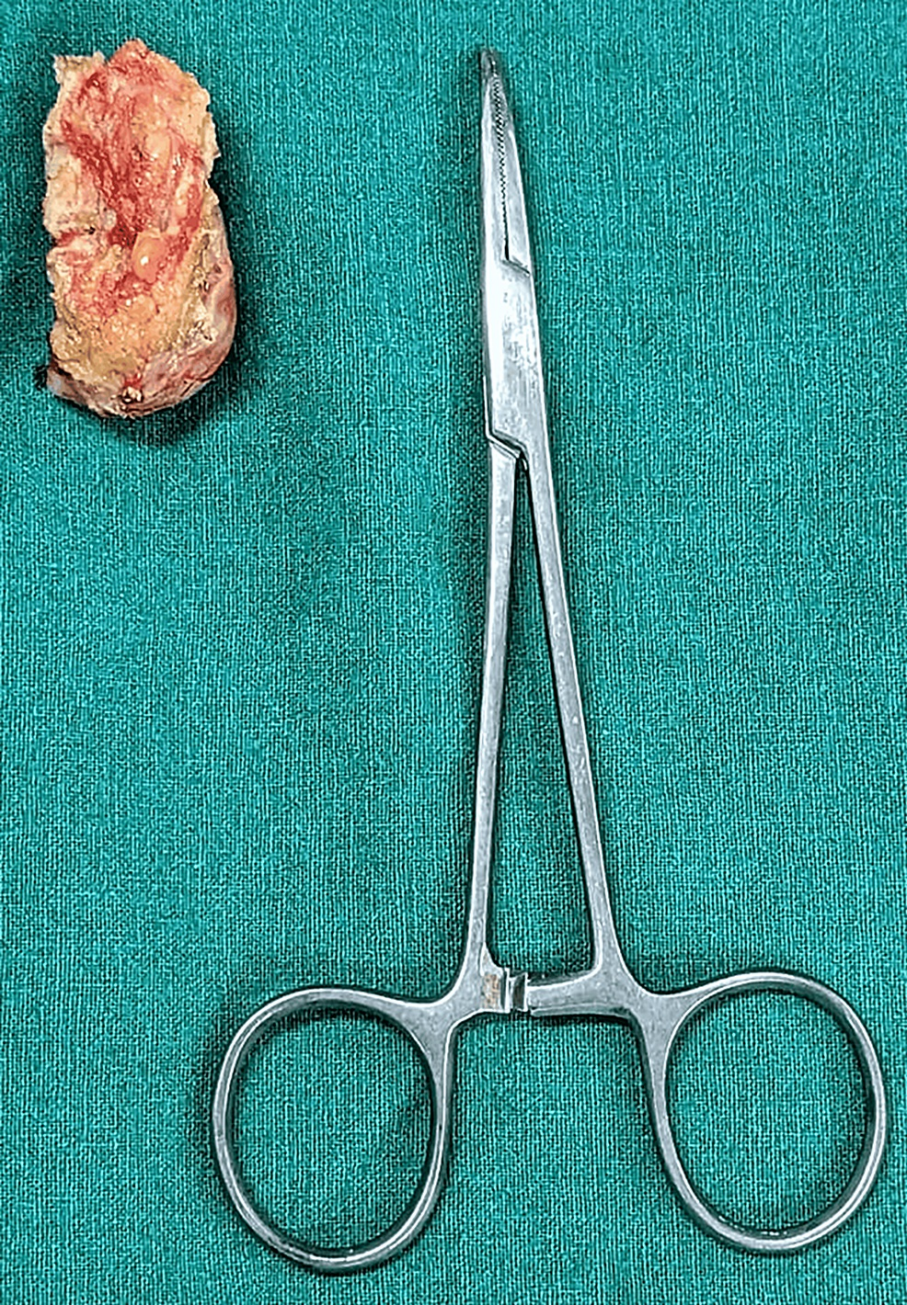
Excised specimen of tumor over back

Subsequently, tumor excision was performed on the patient under general anesthesia. A vertical incision was made on the swelling, initiating dissection along a well-defined plane between the swelling and the surrounding muscles. Feeding vessels were securely tied off, and the wound was closed in layers. The excised mass (Figure 2) was sent for biopsy.

**Figure 3:**
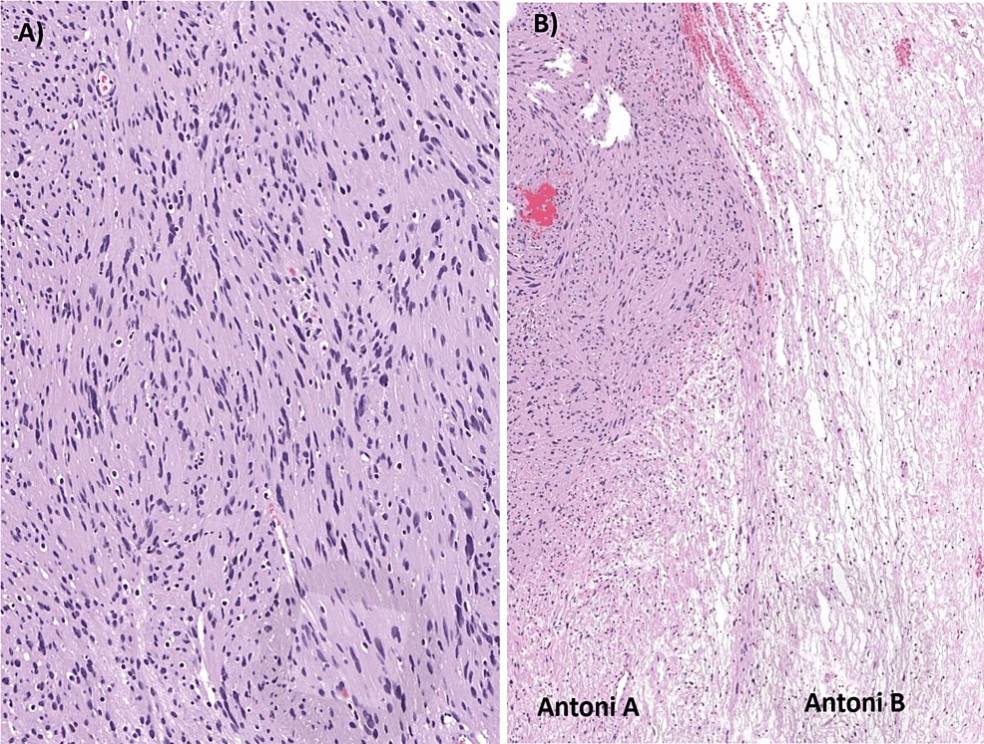
Histopathological image of microscopic schwannoma (A) Verocay bodies; (B) Antoni A area and Antoni B area

The biopsy findings indicated the presence of a capsulated tumor demonstrating a biphasic pattern. The report highlighted the presence of Verocay bodies around the fibrillary process, as illustrated in Figure 3A, with observable nuclear palisading. Upon examination of H&E-stained sections, a compact hypercellular Antoni A area and a myxoid hypocellular Antoni B area were identified (Figure 3B). Within the tissue, collagen fibers and poorly defined cytoplasm were scattered throughout narrow, elongated, and variable cells. Notably, no mitotic figures were visible. In conclusion, the biopsy report suggested that the observed features were consistent with a Schwannoma.

The patient remained stable and was discharged on postoperative day 7. Subsequent follow-ups over the course of one month revealed no symptoms of recurrence, and the patient remained asymptomatic.

## Discussion

Schwannomas, also referred to as neurilemmomas, are rare, typically benign tumors arising from Schwann cells responsible for protecting and maintaining peripheral nerves [3]. Characterized by encapsulation, these tumors exhibit a thin fibrous capsule, contributing to their well-defined and solid nature. While malignancy is uncommon, the encapsulated structure is a distinguishing feature of schwannomas [8]. The anatomical distribution of schwannomas underscores their predilection for the head and neck region, particularly impacting the lateral neck in 25-45% of cases. Manipulation of the tumor may occasionally induce par aesthesia or 'shocks' in the innervated area [5]. Notably, schwannomas presenting as extraspinal swellings over the thoracolumbar region are exceedingly rare, adding a unique dimension to their clinical manifestation [9].

Traditional MRI signs such as the 'split-fat sign' and 'target sign' may be observed, aiding in diagnostic assessments [10]. Degenerative changes like hyalinization, hemorrhage, calcification, and cyst formation can further characterize these tumors [11]. Benign neurogenic tumors, including schwannomas, often exhibit more lateral than longitudinal mobility [12].

In histopathology, spinal schwannomas display characteristic patterns, including Antoni A and Antoni B. Antoni A exhibits Verocay bodies and nuclear palisading, representing a cellular arrangement with significant extracellular matrix and laminin secretion. Antoni B, a loosely organized tissue, may demonstrate myxomatous and cystic alterations [13].

Understanding the histological patterns and radiological features is pivotal for accurate diagnosis and effective management of schwannomas in atypical locations. Surgical excision remains the primary treatment modality, emphasizing the importance of meticulous dissection while preserving surrounding structures. 

## Conclusions

Schwannomas presenting as extraspinal swellings over the thoracolumbar region represent a unique clinical condition. The rarity of such cases in the literature underscores the importance of comprehensive diagnostic approaches, including histopathological and radiological assessments, to determine a differential diagnosis and guide optimal management strategies. 

## References

[1] Koeller KK, Shih RY (2019). Intradural extramedullary spinal neoplasms: radiologic-pathologic correlation. Radiographics.

[2] Safaee MM, Lyon R, Barbaro NM (2017). Neurological outcomes and surgical complications in 221 spinal nerve sheath tumors. J Neurosurg Spine.

[3] Sheikh MM, Jesus OD (2023). Vestibular schwannoma. StatPearls [Internet].

[4] Gangadharan SP (2015). Neurogenic tumors of the posterior mediastinum. Adult Chest Surgery.

[5] Aroori S, Spence RA (2008). Carpal tunnel syndrome. Ulster Med J.

[6] Phulware RH, Sardana R, Chauhan DS, Ahuja A, Bhardwaj M (2022). Extracranial schwannomas of the head and neck: a literature review and audit of diagnosed cases over a period of eight years. Head Neck Pathol.

[7] Kale S, Gosavi V, Jagadale R (2015). Ancient chest wall schwannoma: a case report with review of literature. Med J Dr D Y Patil Univ.

[8] Muramatsu K, Tani Y, Seto T, Iwanaga R, Mihara A, Ihara K, Sakai T (2021). Schwannoma in the extremity: clinical features and microscopic intra-capsular enucleation. J Rural Med.

[9] Gotecha S, Punia P, Patil A (2019). A rare chronic presentation of schwannoma with hemorrhage. Asian J Neurosurg.

[10] Kakkar C, Shetty CM, Koteshwara P, Bajpai S (2015). Telltale signs of peripheral neurogenic tumors on magnetic resonance imaging. Indian J Radiol Imaging.

[11] Malizos K, Ioannou M, Kontogeorgakos V (2013). Ancient schwannoma involving the median nerve: a case report and review of the literature. Strategies Trauma Limb Reconstr.

[12] Behuria S, Rout TK, Pattanayak S (2015). Diagnosis and management of schwannomas originating from the cervical vagus nerve. Ann R Coll Surg Engl.

[13] Joshi R (2012). Learning from eponyms: Jose Verocay and Verocay bodies, Antoni A and B areas, Nils Antoni and schwannomas. Indian Dermatol Online J.

